# A randomized, placebo‐controlled clinical trial of hydrogen/oxygen inhalation for non‐alcoholic fatty liver disease

**DOI:** 10.1111/jcmm.17456

**Published:** 2022-06-23

**Authors:** Geru Tao, Guangjie Zhang, Wei Chen, Chao Yang, Yazhuo Xue, Guohua Song, Shucun Qin

**Affiliations:** ^1^ The Second Affiliated Hospital of Shandong First Medical University Tai'an China; ^2^ Taishan Institute for Hydrogen Biomedical Research Shandong First Medical University & Shandong Academy of Medical Sciences Tai'an China; ^3^ College of Basic Medical Sciences Shandong First Medical University & Shandong Academy of Medical Sciences Jinan China; ^4^ Department of Medical Technology and Nursing Laiwu Vocational and Technical College Jinan China; ^5^ College of Nursing Shandong First Medical University & Shandong Academy of Medical Sciences Tai'an China

**Keywords:** autophagy, clinical trial, MCD‐induced NASH, molecular hydrogen, NAFLD

## Abstract

Non‐alcoholic fatty liver disease (NAFLD) is the most common chronic liver disease worldwide with increasing incidence consistent with obesity, type 2 diabetes and cardiovascular diseases. No approved medication was currently available for NAFLD treatment. Molecular hydrogen (H_2_), an anti‐oxidative, anti‐inflammatory biomedical agent is proved to exhibit therapeutic and preventive effect in various diseases. The purpose of this study was to investigate the effect of hydrogen/oxygen inhalation on NAFLD subjects and explore the mechanism from the perspective of hepatocyte autophagy. We conducted a randomized, placebo‐controlled clinical trial of 13‐week hydrogen/oxygen inhalation (China Clinical Trial Registry [#ChiCTR‐IIR‐16009114]) including 43 subjects. We found that inhalation of hydrogen/oxygen improved serum lipid and liver enzymes. Significantly improved liver fat content detected by ultrasound and CT scans after hydrogen/oxygen inhalation was observed in moderate–severe cases. We also performed an animal experiment based on methionine and choline‐deficient (MCD) diet‐induced mice model to investigate effect of hydrogen on mouse NASH. Hydrogen/oxygen inhalation improved systemic inflammation and liver histology. Promoted autophagy was observed in mice inhaled hydrogen/oxygen and treatment with chloroquine blocked the beneficial effect of hydrogen. Moreover, molecular hydrogen inhibited lipid accumulation in AML‐12 cells. Autophagy induced by palmitic acid (PA) incubation was further promoted by 20% hydrogen incubation. Addition of 3‐methyladenine (3‐MA) partially blocked the inhibitory effect of hydrogen on intracellular lipid accumulation. Collectively, hydrogen/oxygen inhalation alleviated NAFLD in moderate–severe patients. This protective effect of hydrogen was possibly by activating hepatic autophagy.

## 1 INTRODUCTION

Non‐alcoholic fatty liver diseases (NAFLD) which affects 25% worldwide population is the most common chronic liver disease.^1^, ^2^ The disease is etiologically heterogenous affected by genetic and environmental factors and is commonly associated with obesity, insulin resistance, type 2 diabetes mellitus (T2DM), metabolic syndrome (MS) and cardiovascular diseases.^3^ NAFLD was traditionally considered to be a western disease where the prevalence of NAFLD has been continuously increasing due to diet habit and sedentary lifestyle. However, a meta‐analysis published in 2017 demonstrated that the prevalence of NAFLD in Asia is around 25%, similar to western countries and is likely to continue to increase.^4^ NAFLD encompasses a wide histological spectrum of clinical subtypes including simple steatosis (NAFL) and steatohepatitis (NASH).^5^ NAFL is commonly considered as a benign condition, yet its progression can induce further intrahepatic damages.^6^ Approximately 20%–30% NAFL can progress to NASH where inflammation and fibrosis occurs.^7^, ^8^ Among them, 37%–41% NASH patients develop progressive into cirrhosis,^1^ and the incidence of hepatocellular carcinoma (HCC) in NASH patients varies from 2.4% over 7 years to 12.8% over 3 years.^9^ Meanwhile, on account of the common pathogenetic mechanism for NAFLD and MS, patients with NAFLD have higher risk of liver‐related, cardiovascular and all‐cause mortality.^10^ Considering the rising incidence of obesity and diabetes mellitus, NAFLD rises as a serious global health concern in the years to come. Unfortunately, cure of NAFLD is difficult when no FDA‐approved medication is available. Effective lifestyle interventions including dietary adjustment and exercise are proven to be useful,^11^ yet alternative therapeutics are of urge need to prevent disease progression.

In searching for safe and effective therapy of NAFLD, molecular hydrogen, which was proved to be an anti‐oxidative, anti‐inflammatory agent in 2007 was later demonstrated to improve varies types of diseases.^12^, ^13^, ^14^, ^15^ A number of preliminary clinical trials were completed and proved the beneficial effect of molecular hydrogen in more than 70 studies including metabolic syndrome,^16^, ^17^ T2DM,^18^ radiotherapy‐induced injury^19^ and COVID‐19 infection.^20^ In terms of metabolic diseases, that is, NAFLD, molecular hydrogen was proved to be effective in preventing liver fat accumulation in a HepG2 cell model^21^ and animal models.^22^, ^23^, ^24^ A randomized controlled pilot study was reported by Korovljev et al. that hydrogen‐rich water improved mild‐to‐moderate NAFLD.^25^ Though the number of cases in this study was limited, a prospect of molecular hydrogen as an adjuvant treatment is promising. More clinical data are needed to support effectiveness of molecular hydrogen on NAFLD.

Recently, molecular hydrogen was argued as an endogenous regulator of liver homeostasis.^26^ Meanwhile, autophagy, named by its self‐digestive, protective mechanism that involves lysosomal degradation of intracellular organelles is, as well, confirmed to be associated with homeostasis.^27^ Increasing evidence showed that dysregulated autophagy in pathogenesis of a variety of diseases including lipid‐related metabolic disorders.^28^ Furthermore, inducing autophagy is considered as a potential therapeutic strategy in NAFL diseases and fibrosis.^29^, ^30^ Previous studies have reported the impact on autophagy by molecular hydrogen in a variety of animal studies. In a Sprague‐Dawley rat model, molecular hydrogen actives autophagy via HIF‐1α pathways in neuropathic pain.^31^ Promoted autophagy was observed in a chronic intermittent hypoxia induced by renal dysfunction.^32^ In a study of lipopolysaccharide (LPS)‐induced acute lung injury, molecular hydrogen activates autophagy in LPS‐induced rats.^33^ However, whether effect of molecular hydrogen was associated with hepatocyte autophagy in NAFLD was not yet determined.

In this study, we examined the effect of hydrogen/oxygen inhalation on serum lipid level, liver fat deposition in 43 NAFLD patients in a randomized, placebo‐controlled manner. We also applied a diet‐induced NASH model in mice to test the protective effect of hydrogen from histological perspective. Moreover, based on an AML‐12 cell model, we investigated the impact of molecular hydrogen on free fatty acid (FFA)‐induced autophagy.

## 2 MATERIAL AND METHODS

### 2.1 Subjects

This study was a randomized, placebo‐controlled trial, registered at China Clinical Trial Registry (#ChiCTR‐IIR‐16009114). This trial was conducted at Shandong Provincial Coal Taishan Sanatorium and Shandong First Medical University, during May to August 2016. All procedures were conducted in accordance with the Declaration of Helsinki. This trial was approved by The Ethics Committee of Taishan Medical College. All patients signed informed consent to voluntarily participate in this study. Inclusion criteria were as follows: aged 30–70 years old, NAFLD patients meet the diagnosis criteria according to: The diagnosis and management of non‐alcoholic fatty liver diseases: practice guideline by the American Association for the Study of Liver Diseases, American College of Gastroenterology, and the American Gastroenterological Association. Exclusion criteria were as follows: NAFLD caused by other causes, such as tamoxifen; viral hepatitis, liver cirrhosis, drug‐induced injury, autoimmune diseases; alcohol abuse (alcohol consumption >70 g/week for female or >140 g/week for male); patients who are receiving insulin treatment.

Sample size (*n* = 26) was calculated using the power analysis (effect size 0.80, alpha error probability 0.05, power 0.80, non‐sphericity correction Ɛ 2.88) for the primary treatment outcome (G*power 3.1). *Student’s t*‐test with means was used to establish any significant differences found in patients' responses over time of intervention (0 vs. 12 weeks). Sixty‐two patients (aged 58 ± 8 years, 30 women and 32 men) diagnosed with NAFLD according to *Guidelines for management of nonalcoholic fatty liver disease 2010* by Chinese Liver Disease Association were recruited from Zhoudian Community of Tai'an City, Shandong Province, China.

### 2.2 Clinical trial protocol and hydrogen inhalation

All patients were assigned to take general examination including height, body weight, blood pressure and waist circumference, as well as serum test, B‐mode ultrasound examination and computerized tomography (CT) scan before and after the trial. All participants were asked to follow their usual diet, physical activities and lifestyles during the trial. Fasting venous blood samples were drawn for blood tests including total cholesterol (TC), high density lipoprotein cholesterol (HDL‐C), triglyceride (TG) and liver enzymes at the Second Affiliated Hospital of Shandong First Medical University by automatic biochemical analyzer (HITACHI 7080). Serum MDA, SOD (Solarbio), TNF‐α, IL‐6 (Mlbio) quantifications were conducted using commercial kits. Hepatic ultrasonography (USG, Philip Acuson X300) and CT scans (GE Optima CT680) were performed by trained radiologists at Shandong Provincial Coal Taishan Sanatorium blindly before and after the trial. Liver steatosis grade was measured both by USG and calculation of liver/spleen ratio (CT_L/S_) from CT images.

Ultrasonographic diagnostic criteria was as follows. Mild: increased diffused liver echogenicity compared with right kidney, but echogenicity of the intrahepatic vessel walls and diaphragm was well visualized. Moderate: liver echogenicity moderately greater than the right kidney with poor visualization of intrahepatic vessel walls. Severe: significantly increased echogenicity of the liver compared with the right kidney, a lack of visualization of intrahepatic vessel walls, and markedly decreased reflectivity of the hemidiaphragm.^34^

All patients were randomly allocated into 2 groups using a computer‐generated random number table. Thirty patients allocated into hydrogen/oxygen group were assigned to inhale a mixture of 66% hydrogen and 33& oxygen (3 L/min) for 1 h/d. Thirty‐two patients in placebo group were assigned to inhale a mixture of 78.1% nitrogen and 20.9% oxygen (normal air, 3 L/min) for 1 h/d. The intervention lasted for 13 weeks. Hydrogen–oxygen generator and air pumps were provided by Shanghai Asclepius Meditec Co., Ltd.. Both devices used in this experiment have the same appearance with labels covered, so that recruits were not aware of grouping.

### 2.3 Animal experiments protocol

Sixty 8‐week male C57BL6/J mice were purchased from Beijing Huafukang Biotechnology. All animals were maintained under a 12‐h light/12‐h dark cycle in environment of 24 ± 1°C and 55%–65% humidity. Animals were randomly allocated into 6 groups with 10 mice/group: control group (Chow), placebo group (MCD + N_2_ group), hydrogen group (MCD + H_2_ group), pioglitazone group (MCD + PGZ group), hydrogen‐pioglitazone group (MCD + H_2_ + PGZ group) and hydrogen‐chloroquine group (MCD + H_2_ + Chlo group). Except for control group which received a chow diet, all other 5 groups of mice were fed with a methionine and choline‐deficient (MCD) diet that induces NASH.^35^ Animals in MCD + H_2_, MCD + PGZ + H_2_ and MCD + H_2_ + Chlo groups received 1 h/d hydrogen inhalation (66% hydrogen and 33% oxygen), while other groups received 1 h/d nitrogen inhalation (66% nitrogen and 33% oxygen). Animals in MCD + PGZ group and MCD + PGZ + H_2_ group were applied PGZ at 10 mg/kg/day by oral gavage. Animals in MCD + H_2_ + Chlo group were given chloroquine at a dose of 20 mg/kg/d intraperitoneally. All animals were euthanized at week 6 of experiment. Blood and liver samples were collected. Serum was tested for liver function and cytokines. A part of the right posterior lobes of mice were fixed in 4% paraformaldehyde for histological examination and lysed for determination of oxidative stress status and Western blot. H&E staining was conducted for histopathological examination. Liver ROS, MDA and GSH were quantified using commercial kits according to the manufacturers' instruction (ROS: BioLab, Beijing, HR8835, MDA: Solarbio, BC0025, GSH: Solarbio, BC1175). All procedures in animal experiments were conducted in accordance with the guidelines of the Animal Care and Use Committee of Shandong First Medical University.

### 2.4 Reagents and cell culture

Sodium palmitate and sodium oleate were obtained from Sigma‐Aldrich. Stock solution of 50 mM of each reagent was obtained by dissolution in H_2_O at 70°C. Working solutions were made by conjugation of 2 mM palmitic acid or free fatty acid (FFA) mixture (sodium palmitate: sodium oleate = 1:2) with 2% BSA‐MEM/F‐12.^36^ Antibodies including anti‐LC3, anti‐Beclin‐1, anti‐ATG5 and anti‐β‐actin were obtained from Sigma‐Aldrich.

Mouse hepatocyte cell line AML‐12 was obtained from Shanghai Institutes for Biological Science, CAS. Cells were maintained in MEM/F12 medium supplemented with 10% foetal bovine serum, 10 μg/ml insulin, 5.5 μg/ml transferrin, 5 ng/ml selenium and 40 ng/ml dexamethasone. Culture medium containing 0.5% lipoprotein depleted foetal bovine serum (LPDS) was used when FFA was added.

### 2.5 In vitro experiment procedure

AML‐12 cells were seeded in 24‐well cell plates and cultured overnight. Medium was changed with DME/F‐12 containing 0.5% LPDS and additional FFA as indicated. Cell plates of hydrogen‐treated group were maintained in a H_2_ incubator (Wuxi Puhe, PH‐1‐A1) which provides a 5% CO_2_, 20% O_2_, 20% H_2_ and 37°C environment. As the mock group, cell plates were maintained in a cell culture incubator of 5% CO_2_ and 37°C environment. Cells were treated for 12 or 24 h as indicated before harvest for quantification of intracellular TG, Western blot, Oil red O staining and transmission electron microscope (TEM).

### 2.6 Intracellular triglyceride (TG) measurement and oil red O staining

After FFA treatment, cell medium was removed, and cells were washed with PBS for 3 times and lysed with PBS containing 1% Triton X‐100 at room temperature for 20 min. The concentration of TG and total soluble protein were measured using a TG kit (Biosino Bio‐technology & Science Inc.) and a BCA protein assay kit (Solarbio Life Science), respectively. For Oil red O staining, cells were fixed with 4% paraformaldehyde for 20 min before Oil red O staining.^37^

### 2.7 Statistical analysis

Data were represented as mean ± standard deviation (mean ± SD). Comparisons between 2 groups were analysed using Student’s *t*‐test. Difference was considered significant as *, *p* < 0.05; **, *p* < 0.01; ***, *p* < 0.0. All analysis were performed using SPSS 20.0 software (IBM, NY). Figures were designed using Graphpad Prism 7.0 (Graphpad Software).

## 3 RESULTS

### 3.1 Inhalation of hydrogen/oxygen alleviated NAFLD in patients

There were no significant differences regarding age, serum lipids, liver enzymes between the 2 groups of patients at baseline (Table 1). Forty‐three out of 62 patients completed this trial (21 in the placebo group and 22 in the hydrogen/oxygen group) with no side effect or any discomfort reported. Patients who dropped out of intervention also refused to continue assessments and were lost to follow‐up. The compliance with the intervention for placebo and hydrogen/oxygen groups were 68.75% and 70%, respectively.

**TABLE 1:** Demographic characteristics of study participants

Demographic	N group	H_2_ group
Age (year)	53.4 ± 8.2	54.3 ± 7.8
Male patients	14	10
Female patients	8	11
Number of concomitant pathologies		
Diabetes	6 (27.27%)	4 (19.05%)
Hypertension	11 (50%)	7 (33.33%)
Hyperlipidaemia	5 (22.73%)	7 (33.33%)

	N‐Before	N‐After	H_2_‐Before	H_2_‐After
SBP (mmHg)	137 ± 18.7	135 ± 17	138.9 ± 19.4	136.1 ± 16.7
DBP (mmHg)	81.5 ± 11.5	79.7 ± 10	84.3 ± 12.4	80.9 ± 12.3
Weight (kg)	77.4 ± 9.3	77.2 ± 10.5	72.2 ± 8.7	71.6 ± 9.2
WC (cm)	102.3 ± 11.9	100 ± 9.3	95.34 ± 7.95	93.16 ± 7.65
BMI (kg/m^2^)	29.4 ± 2.5	28.9 ± 3.3	27 ± 3	26.6 ± 2.3

*Note*: Data are means ± SD or median or number of subjects.

Abbreviations: BMI, body mass index; DBP, diastolic blood pressure; H_2_, hydrogen/oxygen group; N, placebo group; SBP, systolic blood pressure; WC, waist circumference.

**FIGURE 1:**
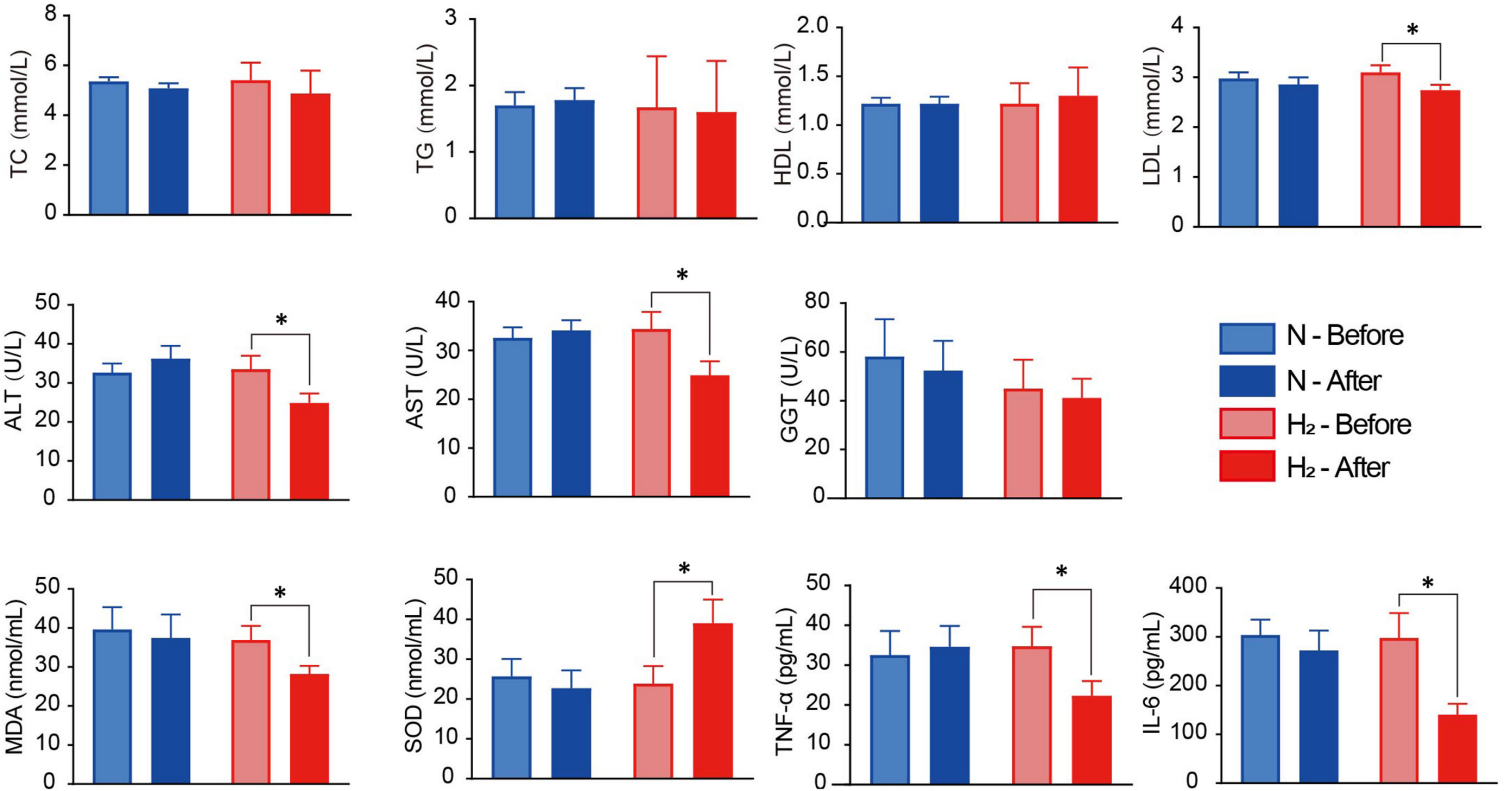
Serum biological, oxidative and immunological index of subjects before and after the trial. Serum samples of subjects in hydrogen/oxygen and placebo groups were collected before and after the trial and analysed at the Second Affiliated Hospital of Shandong First Medical University by automatic biochemical analyser (HITACHI 7080). Serum MDA, SOD, TNF‐α and IL‐6 were quantified using commercial kits. N: placebo group, H_2_: hydrogen/oxygen group. Student's *t*‐test was applied and asterisk * indicates significant difference (*p* < 0.05)

To test whether hydrogen/oxygen inhalation modulates serum lipids, as reported previously in MS patients,^17^ we analysed serum lipids of all patients before and after the trial. TC and TG levels did not show significant changes in both groups after 13 weeks, yet LDL‐C of hydrogen/oxygen group significantly decreased after the trial. More importantly, AST and ALT which are indicators of liver damage decreased in hydrogen/oxygen group indicating a liver protective effect of hydrogen/oxygen inhalation. In addition, biomarkers of oxidative stress and inflammation significantly decreased in hydrogen/oxygen group indicating the systemic anti‐inflammation effect of hydrogen/oxygen gas (Figure 1).

To verify the effect of hydrogen/oxygen inhalation on liver steatosis, USG and CT scan were performed. Alleviated steatosis was found in moderate cases of hydrogen/oxygen group that USG diagnosis and CT_L/S_ value both improved. None of the participants showed normal echogenic responses by USG at baseline. Among mild cases, 16.7% (2/12) in hydrogen/oxygen group and 50.9% (4/8) in the placebo group regressed to normal, and 33.3% (4/12) in hydrogen/oxygen group and 27.5% (3/8) cases in placebo group progressed to moderate cases. Hydrogen/oxygen inhalation exerted no effect in mild cases. However, 77.8% (7/9) of the moderate cases in hydrogen/oxygen group had regressed to mild cases while only 38.5% (5/13) in the placebo group improved. In addition, 15.4% (2/13) of the moderate cases in placebo group progressed to severe cases while none in the hydrogen/oxygen group progressed (Table 2).

**TABLE 2:** Comparison of steatosis grade before and after the trial between hydrogen/oxygen and placebo groups by ultrasound examination

Hydrogen/oxygen group (*n* = 21)	Changes at completion of the trial			
Baseline	Negative	Mild	Moderate	Severe
Mild (*n* = 12) (57.2%)	2/12 (16.7%)	6/12 (50%)	4/12 (33.3%)	0/12 (0%)
Moderate (*n* = 9) (42.8%)	0/9 (0%)	7/9 (77.8%)	2/9 (22.2%)	0/9 (0%)
Severe (*n* = 0) (0%)	0/0 (0%)	0/0 (0%)	0/0 (0%)	0/0 (0%)
Total	2/21 (9.5%)	13/21 (61.9%)	6/21 (28.6%)	0/21 (0%)

Placebo group (*n* = 22)	Changes at completion of the trial			
Baseline	Negative	Mild	Moderate	Severe
Mild (*n* = 8) (36.5%)	4/8 (50%)	1/8 (12.5%)	3/8 (27.5)	0/8 (0%)
Moderate (*n* = 13) (59%)	0/13 (0%)	5/13 (38.5%)	6/13 (46.2%)	2/13 (15.4%)
Severe (*n* = 1) (0.5%)	0/1 (0%)	0/1 (0%)	0/1 (0%)	1/1 (100%)
Total	4/22 (18.2%)	6/22 (27.3%)	9/22 (40.1%)	3/22 (13.6%)

*Note*: Data are number of subjects with percentages in parentheses.

**FIGURE 2:**
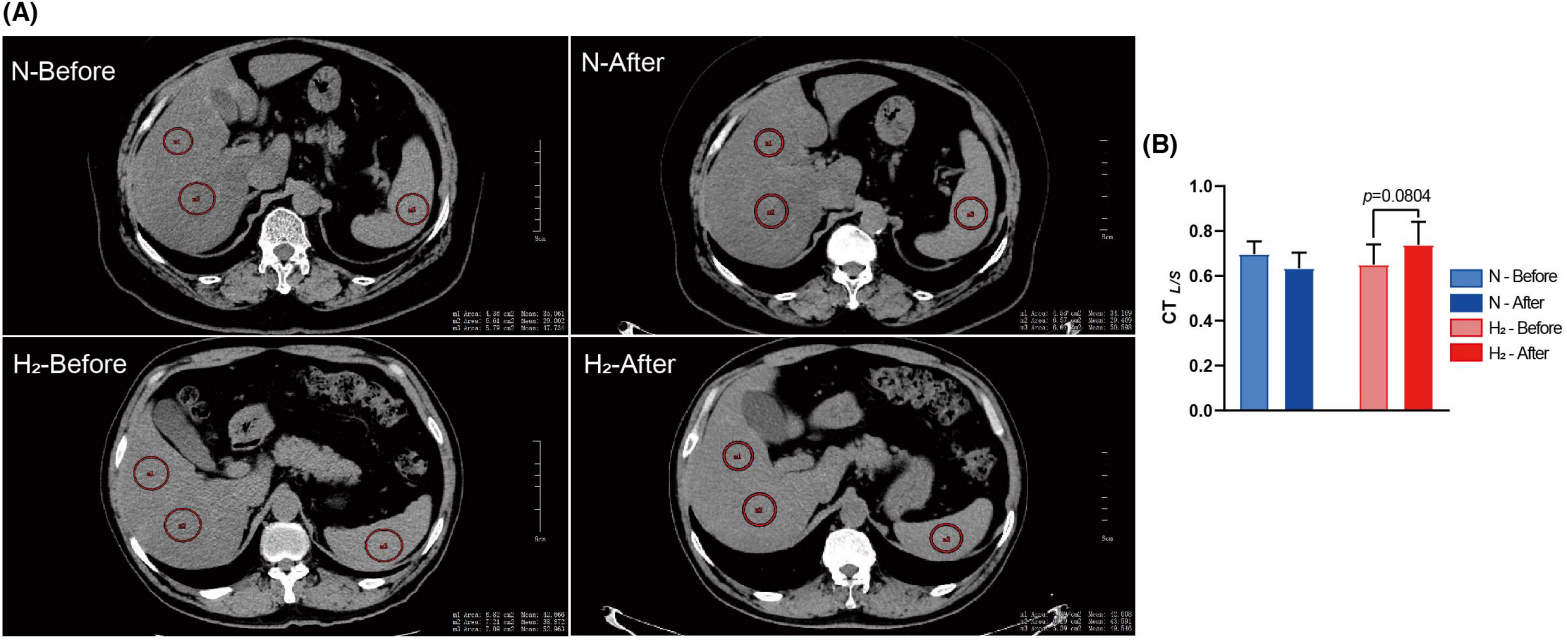
Representative liver CT scan of patients from hydrogen/oxygen and placebo groups. CT scans were conducted in Shandong Provincial Coal Taishan Sanatorium before and after the trial. Images were analysed using Onis Viewer 2.6. Hounsfield units were obtained from 2 region of interest (ROI) in the right lobe of liver and 1 site of the spleen. Liver/spleen CT ratio (CT_*L/S*_) was obtained by calculation of L/S

Before the trial, 13 participants had at least 30% hepatic steatosis on CT (CT_*L/S*_ < 0.8^38^). Among these patients, a trend of overall improvement of CT_*L/S*_ can be seen in hydrogen/oxygen group (*n* = 8) compared with placebo group (*n* = 7) (Figure 2A). Only 28.6% (2/7) participants had improved CT_*L/S*_ in the placebo group after the trial, while 75% (6/8) participants in hydrogen/oxygen group showed alleviated hepatic steatosis. The CT_*L/S*_ after trial in hydrogen/oxygen group was improved than that of the placebo group (Table 3, Figure 2B). CT scan results were consistent with ultrasonography that hydrogen/oxygen inhalation alleviated moderate hepatic steatosis in patients.

**TABLE 3:** Comparison of steatosis grade before and after the trial among patients with 30% or above hepatic steatosis (CT_*L/S*_ < 0.8) between hydrogen/oxygen group and placebo groups by CT

Placebo group (*n* = 7)	N‐Before	N‐After
CT_*L/S*_	0.6977 ± 0.0558	0.6346 ± 0.0694
Difference between before and after the trial	−0.063 ± 0.089	

Hydrogen/oxygen group (n = 8)	H_2_‐Before	H_2_‐After
CT_*L/S*_	0.65 ± 0.09035	0.739 ± 0.1025
Difference between before and after the trial	0.089 ± 0.137	

*Note*: Data are represented as means ± SD.

### 3.2 Hydrogen inhalation alleviated NASH in MCD diet‐induced mice model

**FIGURE 3:**
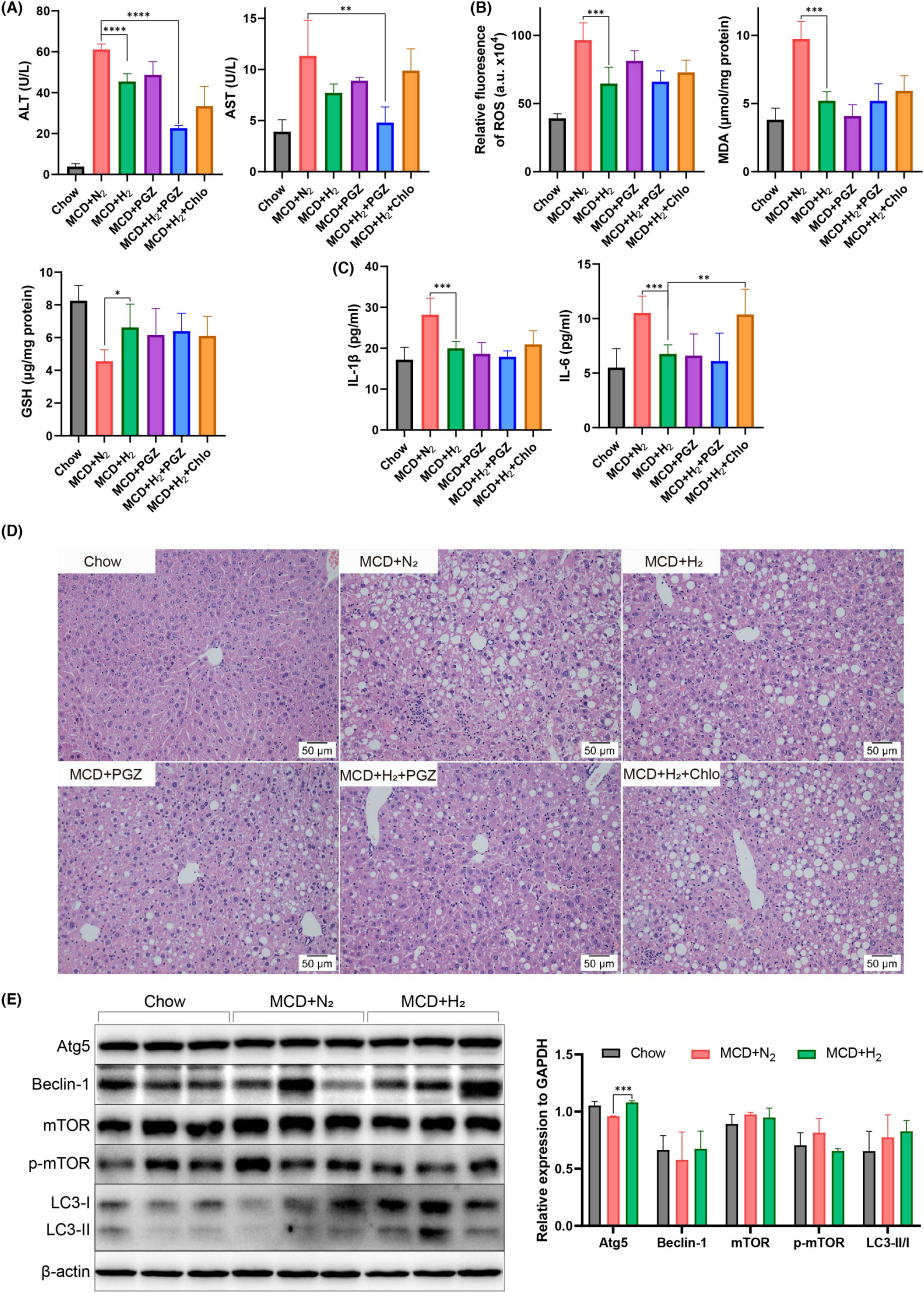
Hydrogen inhalation ameliorates MCD diet‐induced NASH model in C57B6/J mice. Serum ALT, AST (A) and cytokines (C), liver redox status were measured (B). (D) Liver H&E staining was performed, and representative images were shown. (E) Autophagy of mice liver from Chow, MCD + N_2_ and MCD + H_2_ groups were assessed by Western blot. Comparisons were conducted between 2 groups using Students' *t*‐test. * indicates *p* < 0.05, ** indicates *p* < 0.01, ***p < 0.0001

To confirm the beneficial effect of hydrogen gas in animal models, a diet‐induced mouse NASH model was used. C57B6/J mice were fed with MCD diet and inhale 1 h/d 66% hydrogen and 33% oxygen for 6 weeks. Serum cytokines were analysed and liver histological examination was carried out. Blood test showed that ALT in H_2_ group significantly decreased compared with N_2_ group, while AST in H_2_ group slightly decreased (Figure 3A). Increased levels of liver ROS and MDA in N_2_ group significantly decreased in response to hydrogen inhalation, and liver GSH level was improved (Figure 3B). The serum inflammatory parameters were also significantly decreased in hydrogen inhalation group (Figure 3C). Moreover, administration of chloroquine partially blocked the beneficial effect of hydrogen inhalation in AST, liver ROS and MDA and inflammatory cytokines. Combination of PGZ treatment and hydrogen inhalation yielded greater effects than the simple therapeutic intervention in improving AST and ALT suggesting synergism (Figure 3A).

Histopathology examination showed that MCD + N_2_ group developed hepatic micro‐ and macro‐vesicular steatosis and lobular inflammation at 6 weeks. In contrast, hydrogen inhalation alleviated liver histopathology with reduced steatosis and inflammation. In MCD + PGZ and MCD + H_2_ + PGZ groups, hepatic inflammation and steatosis were significantly mitigated. However, in MCD + H_2_ + Chlo group, steatosis and lobular inflammation were observed indicating that chloroquine blocked the beneficial effect of hydrogen. (Figure 3D). Consistent with the serum test results, hydrogen alleviated MCD diet‐induced NASH in decreasing hepatic lipid content, systemic inflammation and liver histology. This mitigation effect was diminished by administration of chloroquine.

To explore the impact of hydrogen inhalation on hepatic autophagy, Western blot of liver samples from MCD + N_2_ and MCD + H_2_ groups were conducted. Results showed that relative expression of autophagy related 5 (Atg5), autophagy‐associated proteins Beclin‐1 and microtubule‐associated protein light chain (LC3) conversion (LC3‐II/LC3‐I) were slightly higher in MCD + H_2_ group, and phospho‐mTOR (Ser2448) were decreased in MCD + H_2_ group indicating the augmented autophagy by hydrogen inhalation (Figure 3E).

### 3.3 Molecular hydrogen decreased lipid accumulation in AML‐12 cell by promoting autophagy

**FIGURE 4:**
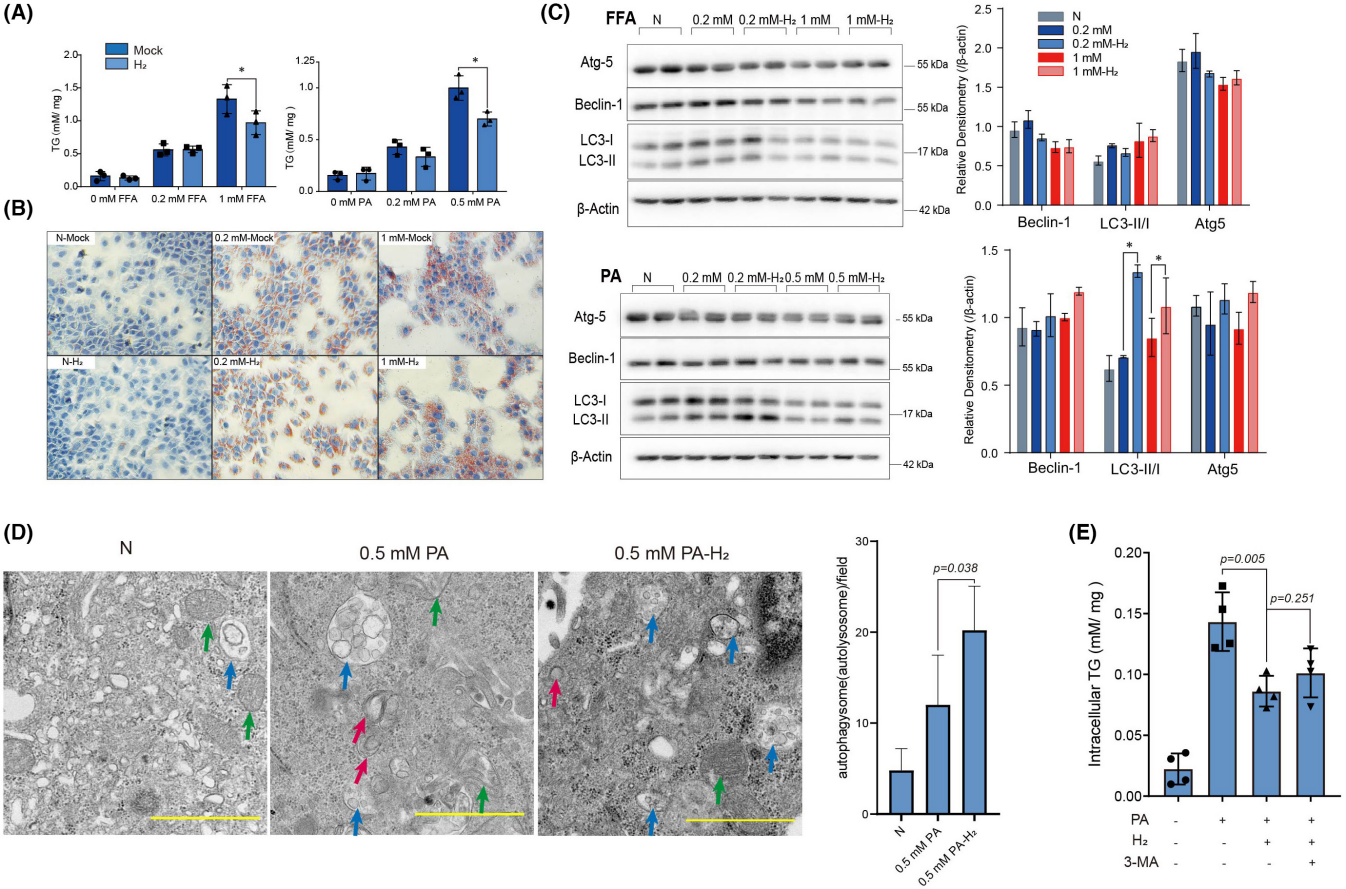
Hydrogen decreased lipid accumulation in AML‐12 cells partially depending on promotion of autophagy. AML‐12 cells were incubated with FFA or PA as indicated and maintained either in conventional CO_2_ incubator (mock) or H_2_ incubator (H_2_) for 24 h. Quantification of TG was normalized to soluble protein by BCA kits (A) and oil red O staining (B). (C) Autophagy of AML‐12 cells incubated with FFA or PA for 12 h was detected by Western blot. (D) Transmission electron microscopy (TEM) of AML‐12 cells exposed to 0.5 mmol/L PA maintained in either CO_2_ incubator or H_2_ incubator for 12 h was conducted. Green, red and blue arrows represent mitochondria, double layered autophagosomes and autolysosomes, respectively. Quantification of autophagic vesicles was shown. Scale bars =1 μm. (E) Intracellular TG quantification of cells incubated with 0.5 mmol/L PA either in CO_2_ incubator or H_2_ incubator for 24 h with additional 3 mmol/L 3‐methyladenine (3‐MA). Statistical analysis was performed using student’s *t*‐test. Asterisk * indicates *p* < 0.05

In order to explore the mechanism of molecular hydrogen in decreasing hepatic lipid content, we used an FFA‐loaded hepatocyte in vitro model. AML‐12 cells were maintained in a 20% H_2_ incubator in the presence of FFA or PA to assess the inhibitory effect of hydrogen on lipid accumulation. Results showed that compared with the mock group which AML‐12 cells were maintained in CO_2_ incubator, intracellular TG was significantly lower in hydrogen‐treated cells when exposed to 1 mmol/L FFA and 0.5 mM PA (Figure 4A,B). Meanwhile, no difference was observed when AML‐12 cells were treated with 0.2 mmol/L FFA or 0.2 mmol/L PA implying that hydrogen exerted no effect on low concentration lipid accumulation.

Next, we investigated the association between hydrogen treatment and autophagy. Western blot of LC3‐II/LC3‐I, Beclin1 and autophagy related 5(Atg5) which take parts in FFA‐induced autophagy were measured. Results demonstrated that hydrogen slightly inhibited autophagy when AML‐12 cells were exposed with 0.2 mmol/L FFA, and slightly promoted autophagy when cells were exposed with 1 mmol/L FFA. The effect of hydrogen on autophagy of FFA‐treated cells were not prominent. However, autophagy was significantly promoted when AML‐12 cells were exposed with PA (Figure 4C).

Since palmitic acid predominantly induces autophagy,^39^ we applied TEM of AML‐12 cells treated with 0.5 mmol/L PA to investigate whether hydrogen treatment caused any ultrastructure change. AML‐12 cells incubated with 0.5 mmol/L PA for 12 h resulted in double layered autophagosomes and a few autolysosomes, while hydrogen‐treated cells developed bigger and more autolysosomes indicating the promoted autophagic (Figure 4D). Moreover, 3‐methyladenine (3‐MA), a specific inhibitor of macro‐autophagy was added to hydrogen‐treated cells to identify whether the inhibitory effect of lipid accumulation by hydrogen was autophagy‐dependent. Results showed that intracellular TG was restored by addition of 3‐MA indicating that blocking autophagy reversed the inhibitory effect on lipid accumulation by hydrogen (Figure 4E). In conclusion, molecular hydrogen inhibited lipid accumulation in AML‐12 cells partially by promoting autophagy.

## 4 DISCUSSION

In this study, we conducted a randomized placebo‐controlled clinical study on protective effect of hydrogen/oxygen inhalation in 43 NAFLD patients. Our results showed that hydrogen/oxygen inhalation improved liver lipid level in moderate–severe cases based on ultrasound analysis. Animal experiments exhibited significant improvement in blood lipid level, inflammation and liver histology. Augmented autophagy by hydrogen treatment was observed both in MCD‐induced mice and AML‐12 cells incubated with PA. In addition, inhibition of lipid accumulation was partially dependent on activated autophagy in vitro. To our knowledge, the study population was relatively larger compared with a previous study^25^ and is the first report describing the impact of molecular hydrogen on hepatocyte autophagy.

In treatment of NAFLD, many pharmaceutical and nonpharmaceutical therapy were tested in clinical trials. Other than lifestyle modification, pioglitazone and vitamin E have been studied the most. Pioglitazone treatment is well documented to improve hepatic steatosis in NASH,^40^, ^41^ yet side effects including weight gain, pedal oedema, bone loss and heart failure are also reported.^42^ Supplementation of Vitamin E is considered as an option in treating non‐cirrhotic non‐diabetic NASH.^43^ However, possible adverse effects including deaths caused by cardiovascular event, hepatotoxicity and increase in bilirubin are uncertain.^43^, ^44^, ^45^ Molecular hydrogen is well documented as a safe agent since high‐pression hydrogen gas is widely used in deep‐diving to prevent decompression sickness and arterial gas thrombi.^46^ In hydrogen biomedicine, most clinical trials reveal no adverse events or side effects, which is also consistent with our study.^47^ An open‐label, prospective, non‐randomized study demonstrated that intravenous administration of hydrogen‐rich solution (200 ml twice a day) is safe for acute cerebral infarction.^48^ Considering molecular hydrogen is a non‐polar covalent, it is understandable that intake of hydrogen causes no toxic effect. However, considering that hydrogen gas is explosive at a range of concentration (4 ~ 75%, v/v), precautions must be taken in clinical use.

A previous report published in 2019 showed that 28‐day intake of hydrogen‐rich water reduces liver fat accumulation evidenced by dual‐echo MRI and serum AST level in mild–moderate NAFLD patients.^25^ Slightly different from this finding, we observed improvement of liver lipid content only in moderate–severe cases. In our clinical trial, four out of 8 mild cases in the placebo group had normal steatosis grade, while only 2 out of 12 patients reached the same result after the trial. We speculated that this might result from lifestyle changes during the trial among recruiters. Another possibility is that the sensitivity of ultrasound in diagnosing mild cases is relatively low compared with moderate cases. Unenhanced CT provides high diagnostic performance in the qualitative diagnosis of macro‐vesicular steatosis of 30% or greater.^38^ We believe data from moderate–severe group was more convincing since placebo‐effects are less pronounced in more severe cases.

Nonetheless, there are several limitations in our study. First, the relatively small cohort of patients being analysed limited subsample analysis for gender difference. Thirteen‐week intervention, though longer than the previous report, was still a short‐time treatment. A study following 24‐week intervention in patients of metabolic syndrome demonstrated that decreased cholesterol and glucose levels, and improved biomarkers of inflammation and redox homeostasis were found in patients drinking hydrogen‐rich water.^49^ We speculated that extended intervention might result in enhanced improvement. Second, the substantial placebo‐effects existing in NAFLD trials should be taken in consideration, especially on short term. We asked the participants not to change their diet and physical activities yet did not monitor whether there is lifestyle modulation during the trial. Third, although the specificity and sensitivity of CT scan in moderate to severe cases are acceptable, chemical shift‐encoded MRI is considered the most accurate and precise method for liver fat quantification.^50^ Accurate assessment of steatosis using CSE MRI should be considered as main criteria in future studies.

In order to investigate the mechanism of molecular hydrogen alleviating hepatic steatosis, we conducted an in vitro study based on an AML‐12 cell model and found that hydrogen treatment promoted PA‐induced autophagy. Regarding to the mechanism of molecular hydrogen alleviating NAFLD, a previous study demonstrated that hydrogen‐rich water alleviated NAFLD by downregulation of miR‐136 through mediating Nrf2 via targeting MEG3. Inhibition of intracellular TG content by hydrogen‐rich medium was blocked when miR‐136 mimic was transfected.^51^ In a diet‐induced NAFLD rat model, intraperitoneally administration of hydrogen‐rich saline activates peroxisome proliferator‐activated receptor (PPAR) α and PPARγ expression in liver, by which hydrogen might suppress fatty acid synthesis and exert anti‐inflammatory effect.^52^ In an in vitro study, molecular hydrogen reduced fatty acid uptake by downregulating CD36, an oxidized low‐density lipoprotein and fatty acid receptor.^21^ Our finding introduced research of molecular hydrogen on autophagy in mitigating NAFLD for further studies.

Regulation of autophagy by molecular hydrogen was also reported in previous studies. In a rat model of vascular dementia, hydrogen suppressed autophagy in the hippocampus possibly by decreasing FoXO1.^53^ In a neuropathic pain study in Sprague‐Dawley rats, hydrogen‐rich saline activated autophagy via HIF‐1α pathways.^31^ Fu et al., reported that hydrogen‐rich saline inhibits lipopolysaccharide‐induced acute lung injury by inhibiting mTOR/TFEB pathway.^33^ Our findings added to a growing body of literature of autophagy regulation by molecular hydrogen.

In summary, current study collected data of effect of molecular hydrogen in mitigating NAFLD from clinical trial, animal experiment and in vitro study. Molecular hydrogen exhibited inhibition effect on liver fat accumulation and promoted autophagy as a protective mechanism. Our findings presented evidence for hydrogen treatment as an adjuvant therapy for NAFLD.

## AUTHOR CONTRIBUTIONS

**Geru Tao:** Data curation (supporting); investigation (equal); writing – original draft (lead); writing – review and editing (lead). **Guangjie Zhang:** Data curation (equal); investigation (equal). **Wei Chen:** Data curation (supporting). **Chao Yang:** Data curation (supporting). **Yazhuo Xue:** Conceptualization (supporting); resources (equal). **Guohua Song:** Conceptualization (equal); resources (equal). **Shucun Qin:** Conceptualization (equal); funding acquisition (equal); writing – review and editing (equal).

## CONFLICT OF INTEREST

The authors declared no conflicts of interest.

## Data Availability

Data are not available due to ethical restrictions.
